# Phase I study to assess the safety and tolerability of olaparib in combination with bevacizumab in patients with advanced solid tumours

**DOI:** 10.1038/bjc.2011.555

**Published:** 2012-01-05

**Authors:** E Dean, M R Middleton, T Pwint, H Swaisland, J Carmichael, P Goodege-Kunwar, M Ranson

**Affiliations:** 1 Clinical Trials Unit, Department of Medical Oncology, The Christie NHS Foundation Trust, The University of Manchester, Wilmslow Road, Manchester M20 4, BX, UK; 2 University Department of Oncology, Oxford NIHR Biomedical Research Centre, Churchill Hospital, Old Road, Headington, Oxford OX3 7LJ, UK; 3 AstraZeneca, Alderley Park, Macclesfield, Cheshire SK10 4TG, UK

**Keywords:** AZD2281, olaparib, bevacizumab, phase 1

## Abstract

**Background:**

Olaparib (AZD2281) is a potent oral poly(ADP-ribose) polymerase inhibitor with anti-tumour activity and acceptable toxicity as monotherapy in patients with BRCA-deficient cancers. The vascular endothelial growth factor receptor inhibitor bevacizumab has been incorporated into standard of care with chemotherapy in various tumours. This phase I study established the safety, tolerability and clinical pharmacokinetics of olaparib alone and in combination with bevacizumab.

**Methods:**

Patients with advanced solid tumours received increasing doses of continuous oral olaparib (100, 200 and 400 mg b.i.d. capsule formulation) in combination with bevacizumab (10 mg kg^−1^ intravenous q2w).

**Results:**

In all, 12 patients enrolled and received treatment. The most common adverse events (AEs) related to olaparib were grade 1/2 nausea and fatigue. No haematological parameters were reported as AEs. No serious AEs related to olaparib or dose-limiting toxicities (DLTs) were reported. Three patients discontinued due to AEs, two patients discontinued both olaparib and bevacizumab and one patient discontinued olaparib. Five patients received combination treatment for over 6 months. There was no evidence that bevacizumab affected olaparib.

**Conclusion:**

The combination of olaparib 400 mg b.i.d. with bevacizumab 10 mg kg^−1^ q2w was generally well tolerated with no DLTs. This combination could be considered for future clinical investigation.

Poly(ADP-ribose) polymerase (PARP) is a protein that contributes to cell survival after DNA damage (reviewed in Virag and Szabo, 2002). After single- and double-DNA strand breaks, the catalytic domains of PARP-1 and PARP-2 are immediately stimulated to critically initiate and regulate DNA damage repair through the base excision repair (BER) pathway. Loss of PARP activity or PARP inhibition leads to an accumulation of single-strand DNA breaks, which after collapse of DNA replication forks, causes double-strand DNA (dsDNA) breaks and tumour cell death. Pre-clinically, cancer cell sensitivity to radiation and DNA-damaging cytotoxics can be enhanced by PARP inhibition (Virag and Szabo, 2002; Nguewa *et al*, 2005). Some tumours have a compromised ability to repair dsDNA breaks by homologous recombination, such as those with mutations in the tumour-suppressor genes *BRCA1* and *BRCA2*. Blockade of BER by PARP inhibition results in an accumulation of dsDNA breaks which are lethal in these cells. This has led to the therapeutic concept of synthetic lethality based on combining two non-lethal events which potently synergise for lethal effect (Bryant *et al*, 2005; Farmer *et al*, 2005; Ashworth, 2008). Germline mutations in the *BRCA1* and *BRCA2* genes account for ∼10% of ovarian and breast cancer cases (Kwon *et al*, 2010). However, in addition, some sporadic tumours share a phenotype similar to familial-*BRCA* cancers due to epigenetic mechanisms of gene inactivation (termed ‘BRCAness’ phenotype) and it is predicted that these patients could potentially derive clinical benefit from PARP inhibition (Turner *et al*, 2004).

Olaparib (AZD2281) is a potent oral PARP inhibitor that has exhibited monotherapy activity in tumour cells with defective components of homologous recombination, including cells with the *BRCA1*^−/−^ and *BRCA2*^−/−^ genotype (Menear *et al*, 2008; Rottenberg *et al*, 2008). Pre-clinical studies have shown that olaparib has the potential to selectively target tumour-specific defects by directly inhibiting PARP-1 and PARP-2, either as monotherapy or in combination with cytotoxic agents. A phase I study of olaparib monotherapy in patients with advanced solid tumours demonstrated that olaparib was well-tolerated and established the maximum tolerated dose (MTD) as 400 mg b.i.d. using a capsule formulation (Fong *et al*, 2009, 2010). Adverse events (AEs) were generally mild and manageable and included nausea and vomiting, fatigue, anorexia, asymptomatic tachycardia, anaemia, neutropaenia and thrombocytopaenia. In this monotherapy study, anti-tumour activity was observed in *BRCA* mutation carriers with 63% clinical benefit and 47% radiological response. Subsequently, olaparib was the first oral PARP inhibitor in phase II clinical trials, and monotherapy activity (400 mg b.i.d.) was demonstrated with acceptable tolerability in patients with advanced breast or ovarian cancers with *BRCA*-related cancer (Audeh *et al*, 2010; Tutt *et al*, 2010). The hypothesis that olaparib, by inhibiting DNA repair, will potentiate DNA damage induced by cytotoxics has been observed in preclinical combination studies. However, four phase I trials of olaparib in combination with topotecan (Samol *et al*, 2011), dacarbazine (Khan *et al*, 2011), paclitaxel (Dent *et al*, 2011) and cisplatin plus gemcitabine (Giaccone *et al*, 2010) showed dose-limiting bone marrow toxicity that was more pronounced than that seen with chemotherapeutic agents alone; the therapeutic gain from these and other combination approaches (including carboplatin and/or paclitaxel, temozolomide, irinotecan or liposomal doxorubicin) are yet to be established.

Bevacizumab is a humanised recombinant antibody that prevents vascular endothelial growth factor (VEGF) receptor binding, inhibiting angiogenesis and tumour growth. Bevacizumab is licensed in combination with chemotherapy in metastatic colorectal cancer (Hurwitz *et al*, 2004), renal cell cancer (Escudier *et al*, 2010) and unresectable advanced non-squamous non-small cell lung cancer (Sandler *et al*, 2006) (licensed doses 5–10 mg kg^−1^ q2w or 7.5–15 mg kg^−1^ q3w). However, initial tumour responses are invariably followed by disease progression reflecting adaptive resistance (Bergers and Hanahan, 2008). One mechanism of resistance is induction of hypoxia as a response to vessel regression caused by the anti-angiogenic agent, resulting in an increase of DNA damage and genetic instability (Chan and Bristow, 2010). The observation that tumour cells exposed to chronic hypoxia acquire defects in homologous recombination and increased sensitivity to PARP inhibition (Hegan *et al*, 2010) is an example of ‘contextual synthetic lethality’ in which hypoxia-induced repair-deficient tumour cells can be targeted by disrupting backup pathways (Chan and Bristow, 2010). Therefore, the premise of combining olaparib and bevacizumab is based on the rationale that direct targeting of PARP by olaparib and indirect sensitisation to olaparib by acquisition of HR defects by bevacizumab will be therapeutically beneficial, and pragmatism as bevacizumab is incorporated into standard-of-care treatments in tumour types that PARP inhibitors have potential to be effective.

The primary objectives of this phase I study were to establish the safety and tolerability of olaparib and bevacizumab combination therapy in patients with advanced solid tumours and to select an appropriate dosing regimen for use in future phase II efficacy studies. Secondary objectives were to compare plasma exposure of olaparib when administered alone and in combination with bevacizumab by assessment of pharmacokinetic (PK) parameters.

## Methods

### Trial design and procedures

This was a two-centre, open-label phase I study evaluating increasing doses of continuous twice daily oral olaparib (100, 200 and 400 mg b.i.d. capsule formulation) in combination with intravenous bevacizumab at a fixed dose (10 mg kg^−1^) administered every 14 days. Patients started olaparib monotherapy as run in for 7 days before combination treatment with bevacizumab at cycle 1. Each treatment cycle lasted 14 days. Bevacizumab was administered 1 h in advance of olaparib initially over 90 min and, if well tolerated, for subsequent cycles over 60 min and then 30 min. All cohorts recruited four patients with planned expansion to six patients in the event of a single dose-limiting toxicity (DLT). To verify dosing compliance and times of olaparib administration, patients were asked to complete a diary card. The end of the study was defined as the date each patient had received a minimum of 6 months treatment.

### Eligibility

Patients aged ⩾18 years with histologically confirmed metastatic cancer not amenable to curative treatments were eligible for this study. Eastern Cooperative Oncology Group (ECOG) performance status 0–2, life expectancy of ⩾12 weeks and adequate bone marrow, liver and kidney function were required. Patients had to have recovered from Common Terminology Criteria for Adverse Events (CTCAEs) grade ⩾2 toxicities (excluding alopecia) caused by previous therapy and were not permitted to have central nervous system metastases, bleeding diathesis or coagulopathy, poorly controlled hypertension, consecutive >+1 proteinuria, recent history of abdominal fistula, gastrointestinal perforation, intra-abdominal abscess, haemorrhage, thrombosis or cardiovascular disease. Written informed consent was obtained from all patients, and this study was approved by an independent ethics committee and conducted in accordance with the Declaration of Helsinki (http://www.ClinicalTrials.gov Identifier NCT00710268).

### Toxicity criteria and dose modifications

Safety assessments included physical examination, haematology and chemistry, electrocardiogram and urinalysis. Toxicities were evaluated at least weekly during the study period and graded using the National Cancer Institute-CTCAE version 3.0. Toxicities were managed by treatment modification or interruption of olaparib until CTCAE grade ⩽1 or resolution. Treatment could then be restarted at 50% dose with a maximum of three permissible dose reductions (providing the dose of olaparib was ⩾50 mg b.i.d.). If the AE failed to resolve during the maximum 28-day interruption period, the patient was withdrawn from the study. No intra-patient dose escalations were permitted. There were no specific dose reductions for the use of bevacizumab, but in the event of toxicity, bevacizumab could be discontinued or temporarily stopped with patients continuing olaparib. A DLT was defined as the following drug-related effects and considered related to olaparib monotherapy during run in or during cycle 1 when in combination with bevacizumab: grade 4 thrombocytopaenia, grade 4 neutropaenia lasting >5 days, grade 3/4 febrile neutropaenia, grade 3/4 nausea and/or vomiting despite maximal anti-emetic therapy and other CTCAE grade 3/4 non-haematological toxicities excluding nausea and vomiting.

### Pharmacokinetics

Venous blood was drawn for determination of PK profiles for olaparib when dosed alone and in combination with bevacizumab. Samples were drawn before dosing and then following the morning dose of olaparib at 30 min, 1, 2, 3, 4, 6, 8 and 12 h on day-7 and day 1 of cycle 1. Plasma olaparib concentrations were determined by high-performance liquid chromatography with tandem mass spectrometric detection. The PK analysis set only included patients who provided both a full steady-state profile of olaparib when administered alone and in combination with bevacizumab. All plasma concentration-time data were analysed with non-compartmental methods using WinNonlin Version 4.1 Enterprise. Pharsight Corporation, Mountain View, CA, USA.

### Statistical methods

There was no formal statistical analysis of safety and tolerability data. Sample size was dependent on tolerability, safety and PK data acquisition while exposing as few patients as possible to study treatment.

## Results

### Patients and extent of exposure

In total, 12 patients (4 in each treatment group) were enrolled and all received at least one dose of olaparib and bevacizumab (Table 1). All 12 patients had previous chemotherapy, 11 had surgery, 5 had radiotherapy, 3 had immunotherapy and 5 had targeted cancer therapies. Patients were heavily pre-treated and 10 had ⩾3 previous chemotherapy regimens. The most common tumour types were colorectal (*n*=4) and breast cancer (*n*=3).

**Figure 1:**
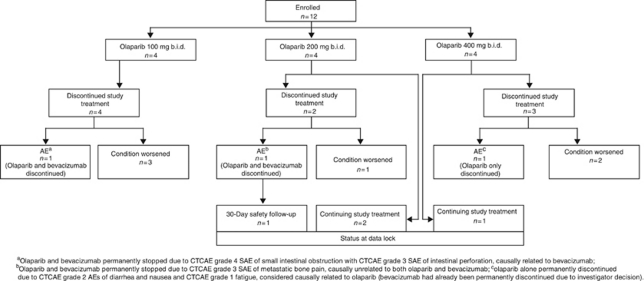
Disposition of all enrolled patients.

All 12 patients completed at least 5 weeks of treatment with olaparib and at least 2 cycles of bevacizumab. At the end of the study, six patients had discontinued treatment due to disease progression, three discontinued due to toxicity and three were still receiving their initial study treatment (Figure 1). Median total treatment duration was 75 days (range 64–103), 217 days (range 161–229) and 69 days (range 42–194) in the 100, 200 and 400 mg groups, respectively. The greater apparent total treatment duration in the 200 mg group was due to the timing of the data cutoff (i.e., dosing in the 200 mg group started ∼1 month earlier than that in the 400 mg group). However, long-term exposure to treatment (>120 days) was tolerated in all patients in the 200 mg group (2 patients received treatment beyond 200 days) and 1 patient in the 400 mg group. Only 1 patient in the 200 mg cohort had a temporary dose interruption of both olaparib and bevacizumab for 21 days due to a serious AE (SAE) of pathological fracture of the left femur (considered unrelated to study treatment). No other patients had dose modifications in either olaparib or bevacizumab.

### Toxicity and safety

No DLTs were observed at any dose during the study. The 12 patients experienced a total of 135 AEs. The most common AEs, reported in three or more patients, are shown in Table 2. Gastrointestinal AEs and fatigue were generally mild to moderate (CTCAE grade 1–2), intermittent and manageable on continued treatment, requiring no modification of study treatment. Nine patients reported AEs that the investigator considered to be possibly related to olaparib (Table 2); nausea (*n*=5) and fatigue (*n*=4) were the most frequently reported. However, seven of the nine patients had AEs that were also considered by the investigator to be possibly related to bevacizumab treatment: all events of dry mouth (*n*=1), fatigue (*n*=4), lethargy (*n*=1), epistaxis (*n*=1), one of two events of mucosal inflammation and one of five events of nausea. In addition, five patients had events that were attributable only to bevacizumab, splinter nail haemorrhage, small intestinal obstruction, intestinal perforation, hypertension, dizziness and anastomotic ulcer (*n*=1 each) and epistaxis (*n*=2).

Six patients had ⩾2 category changes in urinalysis parameters (three patients each with haematuria or proteinuria), none of which were reported as clinically important. There were no clinically relevant changes, or reported AEs, in haematology/clinical chemistry parameters after olaparib treatment alone or in combination compared with pre-dose values. Any observed changes were generally mild transient isolated events requiring no study treatment modification. Six patients reported seven bleeding events during the study; one, three and two patients in the 100, 200 and 400 mg groups, respectively. There were four events of epistaxis and one each of haemoptysis, splinter haemorrhage and haematoma. All bleeding events were mild (CTCAE grade 1) and manageable on continued treatment. The international normalised ratio (INR) was normal in all six patients (none were taking anticoagulants).

Three patients experienced nine events of CTCAE grade ⩾3 (one patient in each dose group; Table 2), none of which were considered by the investigators to be related to olaparib treatment. Four patients experienced a total of eight SAEs (Table 2), none of which were considered to be related to olaparib. In the 100 mg group, a patient had small intestinal obstruction/perforation considered to be related to bevacizumab; a patient in the 200 mg group experienced a pathological fracture of the femur/metastatic pain; one patient in the 400 mg group had a subclavian vein thrombosis and a lower respiratory tract infection; and one patient in the 400 mg group had pyrexia. There were no deaths in the study. Three patients discontinued study treatment due to AEs; two patients discontinued both olaparib and bevacizumab treatment due to SAEs (one patient with metastatic pain unrelated to study treatment and one patient with small intestinal obstruction with perforation considered related to bevacizumab treatment), and one patient discontinued olaparib treatment only due to AEs considered related to olaparib treatment (grade 2 diarrhoea and nausea and grade 1 fatigue; Figure 1).

### Pharmacokinetics

**Figure 2:**
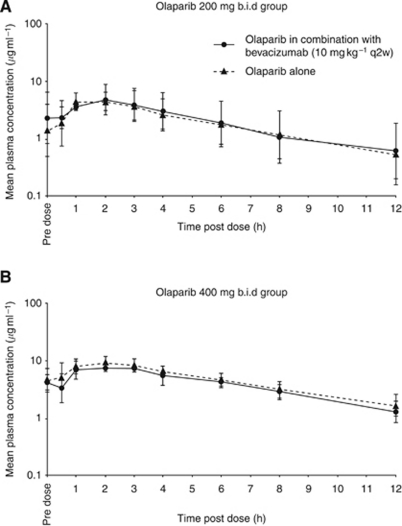
Geometric mean (±s.d.) plasma concentrations of (**A**) olaparib 200 mg b.i.d. and (**B**) olaparib 400 mg b.i.d. alone and in combination with bevacizumab (PK analysis set).

Three patients were excluded from the PK analysis set (two from the 100 mg group and one patient from the 200 mg group) because of blood sampling discrepancies. In addition, for the remaining two patients in the 100 mg group, the time that the second daily dose of olaparib was administered meant that only C_ss,max_, t_max_ and C_ss,max_ ratio data could be reported (Table 3). For both the 200 and 400 mg groups, the Gmean AUC_ss_ for olaparib in combination with bevacizumab was similar to that when administered alone (26.6 compared with 25.8 *μ*g h ml^−1^ for the 200 mg group, and 50.3 compared with 58.1 *μ*g h ml^−1^ for the 400 mg group), with similar inter-patient variability (CV%). Similar results were observed for the Gmean C_ss,max_ (Table 3; Figure 2). For the two patients administered 100 mg olaparib who had available data, one patient had a C_ss,max_ value in combination with bevacizumab that was within 15% of that for olaparib alone; the other patient showed a 40% decrease. The mean ratios of olaparib AUC_ss_ and C_ss,max_ (olaparib with bevacizumab to olaparib alone) for both the 200 and 400 mg groups were both near to 1.0 (1.0 and 0.9 for AUC_ss_ and 1.1 and 0.9 for C_ss,max_, respectively; Table 3). Of the seven assessable patients, four had AUC_ss_ values in combination with bevacizumab that were within 10% of those values for olaparib alone, and three had values within 20%. These results were also reflected in the data for the individual ratios of C_ss,max_. Overall, although based on only a limited number of patients, these data suggest that olaparib exposure is unaffected by co-administration of bevacizumab.

## Discussion

The primary objectives of this phase I study were to establish the safety and tolerability of twice daily olaparib when administered in combination with bevacizumab to patients with advanced solid tumours. In addition, this study aimed to select an appropriate dosing regimen of olaparib in combination with bevacizumab for use in future phase II studies. The MTD of olaparib has previously been determined in patients with solid tumours at 400 mg b.i.d. (Fong *et al*, 2009). In this study, no AEs of CTCAE grade ⩾3, SAEs or DLTs were observed with olaparib, and the dose level of olaparib 400 mg b.i.d. with bevacizumab 10 mg kg^−1^ q2w had an acceptable tolerability profile and could be considered for future clinical studies.

Fatigue, headache and gastrointestinal AEs were most frequently reported in this study, consistent with the safety profile reported in olaparib monotherapy studies. We did not observe myelosuppression as previously published (anaemia 3–17%, thrombocytopaenia 3%, neutropaenia 2–9%) (Fong *et al*, 2009, 2010; Audeh *et al*, 2010; Tutt *et al*, 2010). In monotherapy studies, grade 3/4 haematological toxicities were generally manageable on continued treatment. However, in combination with chemotherapeutics, higher rates of haematological AEs were observed (23–42% neutropaenia (Giaccone *et al*, 2010; Samol *et al*, 2011; Khan *et al*, 2011)), which were refractory to secondary prophylaxis with G-CSF (Dent *et al*, 2011). This suggests that olaparib potentiates cytotoxic-induced myelosuppresion and that overlapping toxicities may restrict future development of some olaparib–cytotoxic combinations. Most of the AEs observed in this study were CTCAE grade 1/2 and no dose reductions were required. Three patients discontinued study treatment due to AEs only, one of which was considered related to olaparib, no further unexpected safety issues were observed with this treatment regimen. Four patients received combination treatment for >6 months but only one patient in the 400 mg group received treatment for >3 months. This suggests long-term tolerability to chronic dosing, but requires further assessment at the recommended phase two dose.

Fatigue and epistaxis were the most frequently reported AEs attributed to bevacizumab. Other observed AEs such as headache, hypertension, dizziness and proteinuria have also been associated with bevacizumab (Genetech, 2011). Mucocutaneous haemorrhage such as epistaxis was observed in four patients and, as in this study, is usually manageable. One patient (100 mg group) with a rectal tumour developed small bowel obstruction on day 104, which subsequently perforated necessitating a laparotomy and segmental resection of the small bowel with end-to-end anastomosis. This SAE was considered possibly related to bevacizumab treatment, which has an incidence of gastrointestinal perforation of 0.3–2.4% (Genetech, 2011). The patient was discontinued from the study and all events resolved post-operatively.

Olaparib is eliminated in humans through the urinary route and faeces, with excretion as a combination of parent compound and metabolites. Metabolism is extensive and, from *in vitro* data, mainly mediated by CYP3A4 (AstraZeneca data on file). The metabolism and elimination of bevacizumab are similar to endogenous IgG, that is, primarily through proteolytic catabolism (Agency, 2011). Although patient numbers are small, as expected based on the known clearance mechanisms for olaparib and bevacizumab, a PK interaction was not observed.

The toxicities of olaparib and bevacizumab were predictable, non-overlapping and the majority were generally manageable with continued dosing. A recent phase I/II trial of olaparib in combination with the VEGF and c-kit inhibitor, cediranib, in patients with recurrent ovarian or metastatic triple-negative breast cancer (but unknown BRCA status) demonstrated haematological DLTs (grade 4 neutropaenia and thrombocytopaenia) and anticipated class toxicities (grade 3/4 neutropaenia, hypertension, fatigue, anorexia, nausea and asymptomatic pulmonary embolism) with an unconfirmed response rate of 56% in ovarian cancer patients (Liu *et al*, 2011). Our study was not designed to assess tumour response, but efficacy of olaparib in combination with VEGF receptor inhibitors alone will need to be demonstrated. One challenge will be identifying a patient population likely to derive benefit, with rational selection based on tumours expressing a ‘mutator’ phenotype due to the acquisition of repair-deficient cancer cells from an unfavourable tumour microenvironment. One patient population that could benefit from this combination treatment are patients with platinum-sensitive recurrent ovarian cancer; recent studies of olaparib (Ledermann *et al*, 2011) and bevacizumab (Aghajanian *et al*, 2011) monotherapy have both demonstrated a significant improvement in progression-free survival. However, although patients who were of BRCA1 or BRCA2 mutations did not seem to have an increased risk of adverse effects in the phase I monotherapy trial (Fong *et al*, 2009), it is plausible that this combination in women with advanced ovarian cancer may yield a higher toxicity profile due to differing disease distribution. Future trials will also need to address how best to integrate olaparib with existing regimens; should olaparib be added to maintenance bevacizumab after first-line chemotherapy or at the emergence of bevacizumab resistance? What is the required duration of olaparib therapy? At present, the olaparib clinical program is focused on identifying sensitive patient subgroups such as *BRCA*-related cancer, to enrich and maximise clinical benefit from this interesting new compound.

In conclusion, olaparib 400 mg b.i.d. in capsule formulation combined with bevacizumab 10 mg kg^−1^ q2w appeared to be a tolerable regimen with no reported DLTs in patients with advanced solid tumours. Future phase II clinical trials of olaparib in combination with bevacizumab, in particular in patients with ovarian cancer, should be considered to assess clinical efficacy and further evaluate the safety and tolerability of this combination.

## Figures and Tables

**Table 1:** Baseline patient demographics and characteristics

	**Olaparib dose (mg b.i.d.)**			
	**100 (*n*=4)**	**200 (*n*=4)**	**400 (*n*=4)**	**All patients (*n*=12)**
*Sex, n (%)*				
Male	1	0	1	2 (17)
Female	3	4	3	10 (83)
Median age (range)	53 (34–65)	51 (22–71)	53 (33–60)	53 (22–71)
				
*Race, n (%)*				
Caucasian	3	4	4	11 (92)
Oriental	1	0	0	1 (8)
				
*ECOG PS at screening, n (%)*				
0	1	3	1	5 (42)
1	3	1	3	7 (58)
				
*Previous therapy n (%)* a				
Radiotherapy	2	1	2	5 (42)
Chemotherapy	4	4	4	12 (100)
Immunotherapy/hormone therapy	0	2	1	3 (25)
Other systemic cancer therapy^b^	2	2	1	5 (42)
Surgery^c^	4	3	4	11 (92)
				
*Primary tumour site, n (%)*				
Colorectal	2	2	0	4 (33)
Breast	2	0	1	3 (25)
Pleura	0	0	1	1 (8)
Skin/soft tissue	0	1	0	1 (8)
Cervix	0	0	1	1 (8)
Pseudomyxoma peritonei	0	0	1	1 (8)
Upper GI	0	1	0	1 (8)

Abbreviations: ECOG=Eastern Cooperative Oncology Group performance status; GI=gastrointestinal.

a Patients may have had more than one previous therapy.

b Patients received previous panitumumab (100 mg group), trastuzumab and lapatinib (100 mg group), cetuximab (200 mg group), AGO14699 (200 mg group) and previous trastuzumab, pertuzumab and lapatinib (400 mg group).

c The most common previous surgical procedures were lymphadenectomy (*n*=3), laparotomy (*n*=2), proctocolectomy (*n*=2), splenectomy (*n*=2), liver operation (*n*=2) and abdominal operation (*n*=2).

**Table 2:** Adverse events, DLTs, serious adverse events

	**Olaparib (mg b.i.d.) Number of patients (events)**			**Total patients (%)**
**Number of patients**	**100 (*n*=4)**	**200 (*n*=4)**	**400 (*n*=4)**	**(*n*=12)**
Total patients with ⩾1 AE	4 (17)	4 (71)	4 (47)	12 (100)
*Adverse events occurring in ⩾3 patients (all causality)*				
Nausea	0	4	3	7 (58)
Constipation	0	3	1	4 (33)
Diarrhoea	0	2	2	4 (33)
Vomiting	0	3	0	3 (25)
Fatigue	1	2	3	6 (50)
Headache	1	2	2	5 (42)
Dizziness	0	1	2	3 (25)
Epistaxis	0	3	1	4 (33)
Oropharyngeal pain	0	3	0	3 (25)
Patients with ⩾1 AE related to olaparib^a^	2 (3)	3 (5)	4 (11)	9 (75)
				
*AEs related to olaparib* a				
Nausea	0	2	3	5 (42)
Fatigue	0	1	3	4 (33)
Mucosal inflammation	1	0	1	2 (17)
Dysgeusia	1	0	1	2 (17)
Diarrhoea	0	0	1	1 (8)
Dry mouth	0	0	1	1 (8)
Dyspepsia	1	0	0	1 (8)
Headache	0	0	1	1 (8)
Lethargy	0	1	0	1 (8)
Epistaxis	0	1	0	1 (8)
Patients with AEs of CTCAE grade ⩾3	1 (3)	1 (5)	1 (1)	3 (25)
				
*AE of CTCAE grade ⩾3*				
Dyspnoea	0	0	1	1 (8)
Bone/flank/extremity/metastatic pain/pathological fracture	0	1	0	1 (8)
Small intestinal obstruction^b^/intestinal obstruction/intestinal perforation^b^	1	0	0	1 (8)
Patients with SAEs	1 (3)	1 (2)	2 (3)	4 (33)
				
*SAEs*				
Pathological fracture/metastatic pain	0	1	0	1 (8)
Subclavian thrombosis	0	0	1	1 (8)
Lower respiratory tract infection	0	0	1	1 (8)
Pyrexia	0	0	1	1 (8)
Small intestinal obstruction^b^/intestinal obstruction/intestinal perforation^b^	1	0	0	1 (8)

Abbreviations: AE=adverse event; CTCAE=Common Terminology Criteria for Adverse Event; DLT=dose-limiting toxicity; SAE=serious adverse event.

a Causation assessed by the investigator.

b Considered by the investigator to be related to bevacizumab treatment.

**Table 3:** Pharmacokinetic parameters of olaparib alone and in combination with bevacizumab

**Olaparib dose (mg b.i.d.)**						
**PK parameter**	**100 (*n*=2)**	**100 + bevacizumab (*n*=2)**	**200 (*n*=3)**	**200 + bevacizumab (*n*=3)**	**400 (*n*=4)**	**400 + bevacizumab (*n*=4)**
C_ss,min,_ *μ*g ml^−1^^a^	N/A	NA	0.5 (191.2)	0.6 (158.6)	1.6 (46.1)	1.3 (43.9)
C_ss,max,_ *μ*g ml^−1^^a^	2.5	1.8	4.7 (35.6)	5.2 (49.0)	9.1 (27.2)	8.1 (17.2)
t_max_, h^b^	2 (2–2)	1 (1–1)	1 (1–2)	2 (1–3)	2 (2–3)	2 (1–3)
AUC_ss_, *μ*g h ml^−1^^a^	NA	NA	25.8 (70.0)	26.6 (78.1)	58.1 (29.4)	50.3 (23.1)
AUC_ss_, ratioc,d	NA	NA	NA	1.0 (12.0)	NA	0.9 (6.1)
C_ss,max_ ratioc,e	NA	0.7	NA	1.1 (22.1)	NA	0.9 (12.2)

Abbreviations: AUC_ss_=area under the plasma concentration–time curve during any dosing interval at steady state; C_ss,max_=maximum plasma (peak) concentration in plasma during dosing interval; C_ss,min_=minimum plasma (trough) concentration in plasma during dosing interval; CV=coefficient of variation; G_mean_=geometric mean; NA=not assessable; PK=pharmacokinetics; t_max_=time to reach peak or maximum concentration of maximum response after drug administration.

a Gmean (CV%).

b Median (range).

c Mean (CV%).

d Ratio of AUC_ss_=AUC_ss_ for olaparib in combination with bevacizumab to AUC_ss_ for olaparib alone.

e Ratio of C_ss,max_=C_ss,max_ for olaparib in combination with bevacizumab to C_ss,max_ for olaparib alone.
